# Maternal Pregnancy Levels of *trans*-Nonachlor and Oxychlordane and Prevalence of Cryptorchidism and Hypospadias in Boys

**DOI:** 10.1289/ehp.1103936

**Published:** 2011-09-07

**Authors:** Britton Trabert, Matthew P. Longnecker, John W. Brock, Mark A. Klebanoff, Katherine A. McGlynn

**Affiliations:** 1 Hormonal and Reproductive Epidemiology Branch, Division of Cancer Epidemiology and Genetics, National Cancer Institute, National Institutes of Health, Department of Health and Human Services, Rockville, Maryland, USA; 2 Epidemiology Branch, National Institute of Environmental Health Sciences, National Institutes of Health, Department of Health and Human Services, Research Triangle Park, North Carolina, USA; 3 National Center for Environmental Health, Centers for Disease Control and Prevention, Atlanta, Georgia, USA; 4 Department of Environmental Studies, and; 5 Department of Chemistry, Warren Wilson College, Asheville, North Carolina, USA; 6 Center for Perinatal Research, The Research Institute, Nationwide Children’s Hospital, Columbus, Ohio, USA

**Keywords:** chlordanes, cryptorchidism, hypospadias, oxychlordane, pregnant women, prospective cohort, *trans*-nonachlor

## Abstract

Background: The etiologies of the male urogenital anomalies—cryptorchidism and hypospadias—are poorly understood. Given positive associations between chlordane isomers and testicular germ cell tumors, it is reasonable to assume that chlordanes might also be associated with other testicular dysgenesis syndrome disorders, namely cryptorchidism and hypospadias.

Objective: To examine whether exposure to *in utero* chlordane is related to cryptorchidism and hypospadias, we evaluated levels of chlordane derivatives, *trans*-nonachlor and oxychlordane, among pregnant women enrolled in the Collaborative Perinatal Project (CPP).

Methods: From 1959 to 1965, the CPP enrolled pregnant women at 12 U.S. medical centers. We analyzed serum *trans*-nonachlor and oxychlordane levels measured in third-trimester serum from the mothers of 217 sons with cryptorchidism, 197 sons with hypospadias, and 557 sons with neither condition. Adjusted odds ratios (ORs) and 95% confidence intervals were calculated using conditional logistic regression.

Results: The quartile-specific ORs for cryptorchidism or hypospadias show no notable associations with *trans*-nonachlor or oxychlordane. Further, there were no significant trends with increasing quartile of maternal *trans*-nonachlor or oxychlordane level in either cryptorchidism or hypospadias (*p*-trend all > 0.45).

Conclusions: The results do not support an association between chlordane levels and cryptorchidism or hypospadias. It is unlikely that current chlordane exposure is related to the development of either anomaly, given that serum chlordane levels at the time of sample collection, the early 1960s, were considerably higher than levels at present.

Chlordane, a human-made chemical mixture of structurally similar organochlorines, was widely used on agricultural crops and as a termiticide in the United States until 1988 (Abadin et al. 1994). Chlordane consists of > 140 isomers; the most abundant include *trans*-chlordane, *cis*-chlordane, *trans*-nonachlor, beta-chlordane, and heptachlor. In humans the predominant chlordane-related contaminants detected are *trans*-nonachlor and oxychlordane, a major metabolite of the chlordanes and nonachlors (Dearth and Hites 1991; Ivie 1973). Chlordanes have a 10- to 20-year half-life in soil, and exposure as a result of termite treatment around dwellings may have continued for decades after application (Bennett et al. 1974).

Chlordanes, specifically nonachlor derivatives (*trans*-nonachlor and *cis*-nonachlor), have been among the organochlorines most consistently associated with testicular germ cell cancer (Cook et al. 2011). Of the four published studies evaluating serum chlordane levels and testicular germ cell tumors (TGCT) (Biggs et al. 2008; Hardell et al. 2003; McGlynn et al. 2009b; Purdue et al. 2009), three reported positive associations with serum nonachlor. One was a retrospective evaluation in Sweden (Hardell et al. 2003), and two were prospective evaluations, one in the United States and one in Norway (McGlynn et al. 2009b; Purdue et al. 2009). In animal experiments, chlordane has been shown to interfere with steroid hormone metabolism, perhaps because of the induction of cytochrome oxidase enzymes (Welch et al. 1971). Given the associations between chlordane isomers and TGCT, it is reasonable to assume that chlordane levels might also be associated with other testicular dysgenesis syndrome disorders, namely cryptorchidism (failure of one or both testicles to descend into the scrotum) and hypospadias (urethral opening on the ventral side of the penis or on the perineum) (Skakkebaek 2003).

Cryptorchidism and hypospadias are common genital birth defects, affecting approximately 3–4% and 0.2–1% of male newborns, respectively. Increases in both congenital malformations have been reported in several but not all countries (Paulozzi 1999; Toppari et al. 2010). Both conditions can be outcomes of androgen insufficiency (Toppari et al. 2010). Shared risk factors for cryptorchidism and hypospadias include premature delivery, intrauterine growth restriction, and concomitant genital abnormalities (Toppari et al. 2010). The etiologic role of lifestyle and environmental exposures such as smoking and organochlorines has been evaluated in some studies, but few have evaluated the association between maternal chlordane levels and either male congenital anomaly. In the only published report to date, Damgaaard et al. (2006) found a positive association between breast milk chlordane levels and cryptorchidism in sons. The paucity of data on the relation of chlordane isomers with male birth defects indicates the need for additional studies.

In a case–control study nested within a large prospective cohort of pregnant women, we evaluated whether levels of *trans*-nonachlor and oxychlordane in maternal third-trimester serum were associated with cryptorchidism or hypospadias among male children.

## Materials and Methods

*Study population.* The Collaborative Perinatal Project (CPP) was a prospective study of neurologic disorders and other conditions in children (Niswander and Gordon 1972). From 1959 to 1965, the study enrolled 48,197 women upon presenting for prenatal care at 12 medical centers in the United States. Centers were located in Baltimore, Maryland; Boston, Massachusetts; Buffalo, New York; Memphis, Tennessee; Minneapolis, Minnesota; New Orleans, Louisiana; New York, New York (two centers); Philadelphia, Pennsylvania; Portland, Oregon; Providence, Rhode Island; and Richmond, Virginia. Eleven centers recruited participants from the prenatal clinics of a university hospital, and one (Buffalo) recruited from 13 private obstetric practices. The study was not intended to be representative of the United States, and each clinical site had its own sampling approach (varying from 10 to 100% of eligible women, either by enrolling a random or systematic sample or all women).

Women were ineligible if they were incarcerated, planned to leave the area after delivery, planned to place their child for adoption, or if they delivered on the day they were recruited for the study. Four percent of the participants were lost to follow-up before delivery. As part of data collection, the mothers were asked to donate nonfasting blood samples at approximately 8-week intervals throughout their pregnancies. Serum samples were stored in glass vials at –20°C with no recorded thaws.

There were 142,130 pregnancies among the 48,197 women, including 54,390 pregnancies prospectively (observed) captured by the CPP. The children were systematically assessed for the presence of birth defects and other outcomes at birth and through 7 years of age, with follow-up completed for approximately 75% of the children born into the study. All mothers provided verbal consent to participate (Hardy 2003). The present study was approved by an institutional review board at the National Institutes of Health.

To evaluate our hypothesis, we used data from a nested case–control study of cryptorchidism and hypospadias among sons within the CPP. Details of the nested case–control study have been described previously (Longnecker et al. 2002). Inclusion criteria were based on the characteristics of the mother and infant. The sole maternal inclusion criterion was the availability of a 3-mL aliquot of third-trimester serum. Inclusion criteria based on the characteristics of the infant included alive at birth, male sex, and singleton birth. Of the 28,444 boys born to mothers enrolled prospectively in the CPP cohort, 6,097 were not eligible for inclusion in the current analysis because there was no maternal 3-mL aliquot of third-trimester serum available (*n* = 5,389), the son was not a singleton (*n* = 441), or the son was not live born (*n* = 267). Among the eligible 22,347 boys, there were 241 cases of cryptorchidism and 214 cases of hypospadias. Five boys had both cryptorchidism and hypospadias and were included in each group for analysis. For comparison, we randomly selected a group of boys (*n* = 599) from the remaining eligible boys without a diagnosis of cryptorchidism and/or hypospadias. Controls were selected so that the control:case ratio would be > 2:1 for each case group.

Hypospadias (urethral opening on the ventral side of the penis) was defined as having a diagnosis any time within the first 7 years of life. The degree of hypospadias was not captured in the medical records. The diagnosis of cryptorchidism (failure of one or both testicles to descend into the scrotum) was made by pediatricians based on serial examinations that included inspection and palpation of the genitalia. We defined cryptorchidism as having had a diagnosis of undescended testis(es) at any time during the first year of life. Boys with undescended testis(es) after the first year of life were not considered cryptorchid because they may have had retractile testes. Among the boys with undescended testicle(s) at birth (*n* = 138), all but one had documented orchiopexy or a subsequent observation of cryptorchidism in at least one of the three subsequent examinations (ages 4 months, 1 year, 7 years). For the remaining boys diagnosed as cryptorchid during the first year of life (*n* = 103), study records indicated that the testicles were descended at birth, suggesting that these boys may have had acquired, rather than congenital, undescended testis (Barthold and Gonzalez 2003). To evaluate whether a relationship with oxychlordane or *trans*-nonachlor varied by type of cryptorchidism, we considered boys with testis(es) descended at birth separately in an additional analysis.

For analyses in CPP, the socioeconomic index was calculated as the mean of three percentile scores: education of head of the household, occupation of head of the household or chief wage earner, and family income. The score used to calculate the percentile for an occupation was based on the percentiles of education and income among persons with the same occupation (Myrianthopoulos and French 1968).

*Laboratory assays.* Serum levels of chlordane (*trans*-nonachlor and oxychlordane) were measured at the Centers for Disease Control and Prevention (CDC) after solid-phase extraction cleanup and dual-column gas chromatography using electron capture (Brock et al. 1996). Serum levels of *p,p*´-dichlorodiphenyldichloroethylene (*p,p*´-DDE) and 11 polychlorinated biphenyls (PCBs) were measured in the same laboratory, and the laboratory methods have been described previously (Longnecker et al. 2002; McGlynn et al. 2009a). Serum cholesterol and triglycerides were measured using standard enzymatic assays.

The between-assay coefficient of variation was 25% for *trans*-nonachlor and 20% for oxychlordane. These were determined at concentrations of 0.52 and 0.57 μg/L (301 batches), respectively. Limits of detection (LODs) were 0.28 μg/L for *trans*-nonachlor and 0.20 μg/L for oxychlordane; 29% of the values for *trans*-nonachlor were below that value, whereas 31% of the values for oxychlordane were below the LOD. For values below the LOD, we used the signal recorded by the instrument, when available, because it is thought that the signals below the instrument’s LOD yield better estimates of true concentration than imputed values (Chevrier et al. 2010). Undetected values were set to missing.

*Statistical analysis.* Of the 1,054 total subjects (241 cryptorchid, 214 hypospadias, 599 controls), 3% were missing data on *trans*-nonachlor. Of those with data on *trans*-nonachlor, 6% were missing data on other covariates, and 5% were also missing data on oxychlordane. A total of 971 (217 cryptorchid, 197 hypospadias, 557 controls) subjects were included in the analysis of *trans*-nonachlor, and a total of 919 (206 cryptorchid, 181 hypospadias, 532 controls) were included in the analysis of oxychlordane.

We categorized *trans*-nonachlor and oxychlordane concentrations according to the quartile distributions in controls, with the lowest quartile serving as the reference category. Odds ratios (ORs) and 95% confidence intervals (CIs) for the association between *trans*-nonachlor or oxychlordane and cryptorchidism or hypospadias were estimated using conditional logistic regression conditioned on study center (12 strata). We assessed a linear trend across quartile categories by including a single independent variable taking the value of the corresponding median of the category. Models were adjusted for serum *p,p*´-DDE as a five-stratum categorical variable, total PCBs as a four-stratum categorical variable, and serum triglycerides and cholesterol as continuous variables. We included serum triglycerides and cholesterol as independent variables in all statistical analyses to account for interindividual variations in serum lipid concentration. The results of analyses that modeled lipid-adjusted *trans*-nonachlor and oxychlordane produced results similar to those presented. We included serum lipids as a covariate in our model rather than using lipid-standardized chlordane concentrations because the latter may be prone to bias, depending on the underlying mechanism of the chlordane–lipid disease association (Gaskins and Schisterman 2009).

All models included *trans*-nonachlor or oxychlordane as the main exposure and were adjusted for total PCBs, *p,p*´-DDE, triglycerides, and cholesterol as *a priori* selected variables. Additional variables were assessed as potential confounders using the change in estimate method (Maldonado and Greenland 1993), starting with all variables in the models with deletion of one by one in a stepwise manner. If, on deletion, the OR for the contrast of the highest-to-lowest chlordane strata or the OR from the trend test changed by ≥ 15%, the factor was considered a confounder and was included in the adjusted analyses. Potential confounding factors included race, maternal age, maternal history of previous live birth, season of birth, socioeconomic index, smoking during pregnancy, and gestational hypertension, as defined in Table 1. Additional potential confounding factors included the categorical variables (yes/no) hyperemesis gravidarum, history of infertility, menstrual cycle irregularity, estrogen use during pregnancy, and progesterone use during pregnancy, as well as the continuous variables age at menarche and weight gain during pregnancy. Socioeconomic index was the only variable that changed the OR by ≥ 15%. We also considered the effects of adjustment for the continuous variables birth weight and placental weight and the categorical variables preterm birth and small-for-gestational-age, even though these were potentially intermediate variables.

**Table 1:** Characteristics of mothers and sons according to case–control status of the son, CPP, 1959–1965.

Characteristic	Cryptorchidism (*n* = 217)	Hypospadias (*n* = 197)	Control (*n* = 557)
Race (%)						
White		57.6		50.8		46.5
Black		41.5		43.7		47.8
Other		0.9		5.6		5.7
Gestation (week)						
Median (IQR)		39 (37–40)		39 (37–41)		39 (38–40)
Preterm birth (%)		19.3		20.5		13.8
Birth weight (g)						
Median (IQR)		3,232 (2,863–3,629)		3,147 (2,722–3,487)		3,260 (2,948–3,600)
Small for gestational age (%)		10.7		19.9		5.0
Maternal age (years)						
Median (IQR)		24 (21–30)		23 (20–29)		22 (20–28)
Previous live births (%)						
0		26.3		31.0		30.3
1		23.0		21.8		22.8
≥ 2		50.7		47.2		46.9
Season of birth (%)						
January–March		24.4		17.3		24.6
April–June		28.1		25.4		24.2
July–September		24.9		29.9		25.9
October–December		22.6		27.4		25.3
Socioeconomic index						
Median (IQR)		4.7 (3.3–6.3)		4.7 (3.0–6.3)		4.7 (3.3–6.0)
Prepregnancy BMI						
Median (IQR)		22.2 (20.3–25.0)		21.8 (19.6–24.2)		22.2 (20.0–24.9)
Gestational hypertension (%)		5.9		6.3		6.2
Maternal smoking status (%)						
None		50.7		52.0		55.0
1–10 cigarettes per day		32.3		27.0		26.7
> 10 cigarettes per day		17.0		20.9		18.4
Study center (%)						
Boston, MA		32.7		27.4		24.6
Buffalo, NY		7.4		4.1		3.8
New Orleans, LA		6.5		4.1		4.7
New York City, NY*a*		2.3		3.6		3.1
Baltimore, MD		5.5		6.1		7.9
Richmond, VA		7.4		6.1		6.1
Minneapolis, MN		3.7		4.1		5.4
New York City, NY*b*		1.4		5.1		7.5
Portland, OR		4.1		5.6		7.2
Philadelphia, PA		15.2		23.9		17.6
Providence, RI		10.6		8.1		5.2
Memphis, TN		3.2		2.0		7.0
IQR, interquartile range (quartiles 1–3). **a**Columbia-Presbyterian Medical Center. **b**New York Medical College.						

We evaluated effect modification by maternal age, race, smoking, prepregnancy body mass index (BMI), previous live births, triglycerides, cholesterol, serum *p,p*´-DDE, total PCBs, gestational hypertension, and socioeconomic index, using the cross-product terms. Variables were coded as defined in Table 1, with the exception of prepregnancy BMI, which was coded as < 25, 25–29.9, ≥ 30 kg/m^2^. We supplemented evaluation of effect modification by categorical variables with more than two categories by comparing the model fit statistics for models with and without the cross-product terms. If the *p*-value associated with the interaction term based on the likelihood ratio test had a value < 0.10, the degree of potential effect modification was further evaluated by examining tables stratified by the potentially modifying factor(s). Statistical significance was set at *p* < 0.05 for main effects based on two-sided tests. Statistical analyses were conducted using SAS statistical software package, version 9.2 (SAS Institute Inc., Cary, NC, USA).

## Results

The distribution of selected demographic and health characteristics of the mothers and sons stratified by study group are provided in Table 1. For both cryptorchid and hypospadias case groups, a higher percentage of case boys than control boys were white, born preterm, and small-for-gestational-age (birth weight < 10th percentile). The distribution of other potential confounding factors according to case–control status has been published previously (Longnecker et al. 2002).

The median maternal serum concentrations of *trans*-nonachlor, oxychlordane, DDE, total PCBs, cholesterol, and triglycerides for each study group are shown in Table 2. The median level of maternal serum triglycerides was slightly lower for hypospadias cases (188 μg/L) compared with cryptorchid cases (204 μg/L) or controls (204 μg/L). The Spearman correlation coefficients of *trans*-nonachlor, oxychlordane, DDE, total PCBs, cholesterol, and triglycerides are provided in Table 3. All of the exposures evaluated were positively correlated, with the strongest correlation between *trans*-nonachlor and oxychlordane (*r* = 0.78), as expected. *trans*-Nonachlor was moderately associated with DDE (*r* = 0.52), while the remaining compounds were less correlated (*r* = 0.08–0.46).

**Table 2:** Maternal serum values by son’s case–control status, CPP, 1959–1965 [median (IQR)].

Characteristic	Cryptorchidism (*n* = 217)	Hypospadias (*n* = 197)	Control (*n* = 557)
*trans*-Nonachlor (μg/L)		0.36 (0.26–0.52)		0.40 (0.24–0.58)		0.38 (0.25–0.57)
Oxychlordane*a* (μg/L)		0.29 (0.14–0.47)		0.34 (0.18–0.58)		0.31 (0.16–0.55)
DDE (μg/L)		23.6 (15.9–35.3)		23.8 (16.6–34.4)		24.5 (16.7–37.5)
Total PCBs with imputed congener (μg/L)		2.8 (2.0–3.9)		2.9 (2.1–4.2)		2.7 (1.8–3.9)
Total cholesterol (μg/L)		232 (190–273)		232 (192–277)		234 (197–280)
Triglycerides (μg/L)		204 (161–252)		188 (156–256)		204 (159–259)
IQR, interquartile range (quartiles 1–3). **a**Eleven cases of cryptorchidism, 16 cases of hypospadias, and 25 controls were missing data for oxychlordane, respectively.						

**Table 3:** Spearman correlation coefficients of selected covariates, CPP, 1959–1965.

*trans*-Nonachlor	Oxychlordane	DDE	Total PCBs	Total cholesterol	Triglycerides
*trans*-Nonachlor (μg/L)		1.00										
Oxychlordane (μg/L)		0.78		1.00								
DDE (μg/L)		0.52		0.38		1.00						
Total PCBs (μg/L)		0.38		0.46		0.37		1.00				
Total cholesterol (μg/L)		0.10		0.12		0.15		0.25		1.00		
Triglycerides (μg/L)		0.11		0.12		0.08		0.15		0.36		1.00

Table 4 provides the adjusted ORs for *trans*-nonachlor and oxychlordane quartiles conditioned on study center and adjusted for serum DDE concentration, total PCBs, triglycerides, cholesterol, and socioeconomic index. For cryptorchidism, the quartile-specific ORs for oxychlordane or *trans*-nonachlor were close to the null as well as not statistically significant. Similarly for hypospadias, the quartile-specific ORs for *trans*-nonachlor and oxychlordane were close to the null as well as not statistically significant. We found no significant increase or decrease in risk of either cryptorchidism or hypospadias with increasing quartile of maternal *trans*-nonachlor or oxychlordane level (trend *p*-values all > 0.40).

**Table 4:** Adjusted ORs and 95% CIs for cryptorchidism and hypospadias in relation to maternal oxychlordane and *trans*-nonachlor levels (μg/L), CPP, 1959–1965.

Cryptorchidism					Hypospadias				
Chlordane (quartiles)*a*	Control (*n*)	Case (*n*)	Unadjusted OR	Adjusted OR*b* (95% CI)	Case (*n*)	Unadjusted OR	Adjusted OR*b* (95% CI)
*trans*-Nonachlor (µg/L)														
0.00 to < 0.25		134		47		1.00 (reference)		1.00 (reference)		50		1.00 (reference)		1.00 (reference)
0.25 to < 0.38		139		67		1.25		1.27 (0.85, 1.90)		44		0.83		0.84 (0.54, 1.33)
0.38 to < 0.57		142		62		1.33		1.46 (0.93, 2.30)		52		1.00		1.04 (0.64, 1.70)
≥ 0.57		142		41		1.10		1.22 (0.70, 2.12)		51		0.99		1.08 (0.62, 1.89)
Trend test*c*		557		217				0.55		197				0.60
Oxychlordane (µg/L)														
0.00 to < 0.16		131		54		1.00 (reference)		1.00 (reference)		38		1.00 (reference)		1.00 (reference)
0.16 to < 0.31		131		56		0.95		0.90 (0.60, 1.35)		47		1.12		1.06 (0.66, 1.69)
0.31 to < 0.55		137		51		0.90		0.91 (0.59, 1.42)		44		1.03		1.07 (0.64, 1.80)
≥ 0.55		133		45		0.94		0.95 (0.55, 1.64)		52		1.15		1.24 (0.69, 2.22)
Trend test*c*		532		206				0.90		181				0.46
**a**Quartile cut points were defined based on 557 and 532 controls for *trans*-nonachlor and oxychlordane, respectively. **b**Adjusted ORs and 95% CIs are from conditional logistic regression models adjusted for serum DDE concentration (five categories), total PCBs (four categories), triglycerides, cholesterol, and socioeconomic index.** c**Ordinal test across four categories using the median value within each group.														

Potential effect modification at a *p*-value of < 0.10 was present for the cryptorchidism–oxychlordane association by smoking, the hypospadias–oxychlordane association by BMI, and the hypospadias–*trans*-nonachlor association by smoking. After further evaluation in stratified models, there were some differences in ORs by smoking or BMI; however, all estimates were consistent with no association. Given that the main effects were not indicative of an association, the results from the stratified models are not presented.

In subanalyses, we evaluated the associations between the chlordanes and cryptorchidism and hypospadias among women with no history of a live birth. We hypothesized that the *trans*-nonachlor and oxychlordane exposure to the male fetus would be highest in this group of women. Although the quartile-specific ORs were increased slightly compared with the corresponding ORs for all women (data not shown), there were no significant trends with increasing quartile of maternal *trans*-nonachlor or oxychlordane level for either cryptorchidism (*trans*-nonachlor: *p*-trend = 0.75; oxychlordane: *p*-trend = 0.97) or hypospadias (*trans*-nonachlor: *p*-trend = 0.68; oxychlordane: *p*-trend = 0.90). Finally, after exclusion of the boys (*n* = 103) whose testicles were initially descended at birth, the association between chlordane level and cryptorchidism was not substantially different from the analysis that included all boys with cryptorchidism (results not shown).

## Discussion

In the present study, a prospective evaluation of chlordane levels during pregnancy and cryptorchidism or hypospadias, the results do not support an association between chlordane levels and cryptorchidism or hypospadias. Further, the lack of association was consistent in the subgroup of primiparous women. The median serum concentration of *trans*-nonachlor in the United States was 14.8 ng/g lipid in 2003–2004, the most recent years for which data are available; the median of oxychlordane was 10.3 ng/g lipid (CDC 2011). The median serum concentrations of *trans*-nonachlor and oxychlordane were 48.0 ng/g lipid and 37.5 ng/g lipid, respectively, in the present study samples that were collected in the 1960s. This suggests that current low-level chlordane exposure is unlikely to be related to the development of either condition.

To our knowledge, this is the first epidemiologic evaluation of the association between chlordane levels and hypospadias and only the second such study of the association between chlordane levels and cryptorchidism. Using breast milk as a proxy for maternal exposure, Damgaard et al. (2006) evaluated persistent pesticide concentrations in milk samples collected from mothers of cryptorchid boys (*n* = 62) and healthy boys (*n* = 68). Maternal milk levels of *trans*-chlordane were slightly higher in mothers of cryptorchid boys than mothers of healthy boys; however, the overall exposure was very low (0.04 ng/g lipid in controls) (Damgaard et al. 2006). The investigators reported no relationship between oxychlordane and cryptorchidism and a borderline significant association between *cis*-chlordane and cryptorchidism. However, the percent detection for *cis*-chlordane was only 30.8%, so median concentrations were not reported (Damgaard et al. 2006). Our finding of no association between oxychlordane level and cryptorchidism is consistent with that of Damgaard and colleagues. We did not evaluate *trans*- or *cis*-chlordane; however, we report no association between *trans*-nonachlor, another chlordane derivative, and cryptorchidism.

Teratogenic effects of chlordane exposure have not been observed in animal studies (Abadin et al. 1994). However, chlordane exposures has been shown to affect reproduction in test animals, delaying puberty, disrupting estrous cycling in females, and reducing fertility by as much as 50% (Welch et al. 1971). Furthermore, chlordane is classified as potentially carcinogenic to humans (Group 2B) by the International Agency for Research on Cancer (IARC 2001) and has been associated with risk of TGCT (Hardell et al. 2003; McGlynn et al. 2009b; Purdue et al. 2009). Although chlordane may be a carcinogen, both animal data and the present study suggest it is not a teratogen.

The present study has several strengths. The major advantages were that the study was a large and prospective evaluation of chlordane exposure and congenital malformations. This study has several potential weaknesses that merit consideration. Chlordane levels were measured on samples stored for approximately 40 years. However, prior studies of freeze–thaw cycles have demonstrated that chlordane levels are quite stable over time (Becker et al. 1997). In addition, cholesterol and triglyceride levels were in the expected range, suggesting that substantial degradation had not occurred. The chlordane concentrations for this study were measured in third-trimester samples; the critical window of exposure might be earlier for hypospadias, likely mid-to-late first trimester (Husmann 2002). However, third-trimester assays should reflect first-trimester exposure, given the long biological half-life of chlordane (Abadin et al. 1994). In the CPP, the prevalence of cryptorchidism and hypospadias among males within the first year of life was 56 per 10,000 and 41 per 10,000, respectively (Myrianthopoulos and Chung 1974). These prevalences were higher than in other U.S. populations (cryptorchidism: 19 per 10,000; hypospadias: 21 per 10,000) (Paulozzi 1999). This difference was likely the result of reduced misclassification in CPP, given that congenital anomalies were identified in a series of systematic examinations, whereas birth defect registries are typically based on routine reports and records only. The ratio of hypospadias to cryptorchidism was somewhat higher than what might be expected. We included hypospadias diagnosed up until 7 years of age, whereas we included cryptorchidism cases diagnosed through the first year of life only. Further, the medical records did not denote the degree of hypospadias, which limited our ability to evaluate the relationship of exposure and disease severity.

In conclusion, these results provide no clear evidence of an effect of *in utero* chlordane (*trans*-nonachlor or oxychlordane) levels on cryptorchidism or hypospadias, and the findings are in line with results from animal studies. Further, it is unlikely that current chlordane exposure is related to the development of either anomaly, because serum chlordane levels at the time of sample collection, the early 1960s, were considerably higher than levels at present.

## References

[r1] Abadin HG, Baynes R, Goetchius PF (1994). Toxicological Profile for Chlordane.

[r2] Barthold JS, Gonzalez R (2003). The epidemiology of congenital cryptorchidism, testicular ascent and orchiopexy.. J Urol.

[r3] Becker PR, Mackey EA, Demiralp R, Schantz MM, Koster BJ, Wise SA (1997). Concentrations of chlorinated hydrocarbons and trace elements in marine mammal tissues archived in the U.S. National Biomonitoring Specimen Bank.. Chemosphere.

[r4] Bennett GW, Ballee DL, Hall RC, Fahey JE, Butts WL, Osmun JV (1974). Persistence and distribution of chlordane and dieldrin applied as termiticides.. Bull Environ Contam Toxicol.

[r5] Biggs ML, Davis MD, Eaton DL, Weiss NS, Barr DB, Doody DR (2008). Serum organochlorine pesticide residues and risk of testicular germ cell carcinoma: a population-based case-control study.. Cancer Epidemiol Biomarkers Prev.

[r6] Brock JW, Burse VW, Ashley DL, Najam AR, Green VE, Korver MP (1996). An improved analysis for chlorinated pesticides and polychlorinated biphenyls (PCBs) in human and bovine sera using solid-phase extraction.. J Anal Toxicol.

[r7] CDC (Centers for Disease Control and Prevention) (2011). Fourth National Report on Human Exposure to Environmental Chemicals. NCEH Pub No. 01-0164:1-6.

[r8] Chevrier J, Harley KG, Bradman A, Gharbi M, Sjodin A, Eskenazi B (2010). Polybrominated diphenyl ether (PBDE) flame retardants and thyroid hormone during pregnancy.. Environ Health Perspect.

[r9] Cook MB, Trabert B, McGlynn KA (2011). Organochlorine compounds and testicular dysgenesis syndrome: human data.. Int J Androl.

[r10] Damgaard IN, Skakkebaek NE, Toppari J, Virtanen HE, Shen H, Schramm KW (2006). Persistent pesticides in human breast milk and cryptorchidism.. Environ Health Perspect.

[r11] Dearth MA, Hites RA (1991). Chlordane accumulation in people.. Environ Sci Technol.

[r12] Gaskins AJ, Schisterman EF (2009). The effect of lipid adjustment on the analysis of environmental contaminants and the outcome of human health risks.. Methods Mol Biol.

[r13] Hardell L, Van BB, Lindstrom G, Carlberg M, Dreifaldt AC, Wijkstrom H (2003). Increased concentrations of polychlorinated biphenyls, hexachlorobenzene, and chlordanes in mothers of men with testicular cancer.. Environ Health Perspect.

[r14] Hardy JB (2003). The Collaborative Perinatal Project: lessons and legacy.. Ann Epidemiol.

[r15] Husmann DA (2002). Micropenis: an animal model and its human correlates.. Adv Exp Med Biol.

[r16] IARC (International Agency for Research on Cancer) (2001).

[r17] Ivie GW (1973). Nature and toxicity of two oxychlordane photoisomers.. J Agric Food Chem.

[r18] Longnecker MP, Klebanoff MA, Brock JW, Zhou H, Gray KA, Needham LL (2002). Maternal serum level of 1,1-dichloro-2,2-bis(p-chlorophenyl)ethylene and risk of cryptorchidism, hypospadias, and polythelia among male offspring.. Am J Epidemiol.

[r19] Maldonado G, Greenland S. (1993). Simulation study of confounder-selection strategies.. Am J Epidemiol.

[r20] McGlynn KA, Guo X, Graubard BI, Brock JW, Klebanoff MA, Longnecker MP (2009a). Maternal pregnancy levels of polychlorinated biphenyls and risk of hypospadias and cryptorchidism in male offspring.. Environ Health Perspect.

[r21] McGlynn KA, Quraishi SM, Graubard BI, Weber JP, Rubertone MV, Erickson RL (2009b). Polychlorinated biphenyls and risk of testicular germ cell tumors.. Cancer Res.

[r22] Myrianthopoulos NC, Chung CS (1974). Congenital malformations in singletons: epidemiologic survey. Report from the Collaborative Perinatal project.. Birth Defects Orig Artic Ser.

[r23] Myrianthopoulos NC, French KS (1968). An application of the U.S. Bureau of the Census socioeconomic index to a large, diversified patient population.. Soc Sci Med.

[r24] Niswander KR, Gordon M (1972). The Women and Their Pregnancies.

[r25] Paulozzi LJ (1999). International trends in rates of hypospadias and cryptorchidism.. Environ Health Perspect.

[r26] Purdue MP, Engel LS, Langseth H, Needham LL, Andersen A, Barr DB (2009). Prediagnostic serum concentrations of organochlorine compounds and risk of testicular germ cell tumors.. Environ Health Perspect.

[r27] Skakkebaek NE (2003). Testicular dysgenesis syndrome.. Horm Res.

[r28] Toppari J, Virtanen HE, Main KM, Skakkebaek NE (2010). Cryptorchidism and hypospadias as a sign of testicular dysgenesis syndrome (TDS): environmental connection.. Birth Defects Res A Clin Mol Teratol.

[r29] Welch RM, Levin W, Kuntzman R, Jacobson M, Conney AH (1971). Effect of halogenated hydrocarbon insecticides on the metabolism and uterotropic action of estrogens in rats and mice.. Toxicol Appl Pharmacol.

